# Egyptian evidence -based pediatric clinical practice adapted guidelines for management of [1] steroid sensitive nephrotic syndrome (EPG/SSNS 2022)

**DOI:** 10.1186/s43054-022-00119-w

**Published:** 2023-02-27

**Authors:** Bahia Moustafa, Mahmoud M. El-Kersh, Sherin Shalaby, Nancy Abdel Salam, Sawsan Moselhy, Gamal Taha Soliman, Abeer Selim, Yasser S Amer, Ashraf Abdel Baky, On Behalf of the Egyptian Pediatric Clinical Practice Guidelines (EPG) Committee

**Affiliations:** 1 Department of Pediatrics, Pediatric Nephrology Division, Faculty of Medicine, Cairo University, Cairo, Egypt; 2 Department of Pediatrics, Pediatric Nephrology Division, Faculty of Medicine, Alexandria University, Alexandria, Egypt; 3 Department of Pediatrics, Pediatric Nephrology Division, Faculty of Medicine, Suez Canal University, Ismailia, Egypt; 4 Department of Pediatrics, Pediatric Nephrology Division, Faculty of Medicine, Ain Shams University, Cairo, Egypt; 5 Department of Pediatrics, Faculty of Medicine, Port Saeed University, Port Fuad, Egypt; 6 Department of Pediatrics, Medical Research and Clinical Studies Institute, National Research Centre, Cairo, Egypt; 7 Alexandria Center for Evidence-Based Clinical Practice Guidelines, Faculty of Medicine, Alexandria University, Alexandria, Egypt; 8 Department of Pediatrics, CPGs & Quality Research Unit, Quality Management Department, and Research Chair for Evidence-Based Health Care and Knowledge Translation, University Medical City, King Saud University, Riyadh, Saudi Arabia; 9 Department of Pediatrics, Pediatric Allergy, Immunology and Rheumatology Unit, Faculty of Medicine, Ain Shams University, Cairo, Egypt; 10 Chairman, Egyptian Pediatric National Clinical Practice Guidelines, Committee, Cairo, Egypt

**Keywords:** Steroids sensitive nephrotic syndrome, Diagnosis, Treatment, Follow-up, Pediatric guidelines

## Abstract

**Background:**

Nephrotic syndrome is one of the most common chronic kidney diseases in children. Steroid sensitive type constitutes about 90% and steroid resistant 10% of total cases.

**Objectives:**

These national adapted guidelines aim to frame evidence-based recommendations adopted or adapted from IPNA 2020, KDIGO 2021, and Japanese 2014 for diagnosis, evaluation, management and follow-up of nephrotic children for Steroid sensitive nephrotic syndrome (SSNS) as paper one to be followed with SRNS as paper two.

**Methodology:**

Formulation of key questions was followed with review of literature, guided by our retrieved and appraised guidelines using Agree plus appraisal tool. After virtual monthly meetings through the year 2021, the final draft was validated considering the comments of external local reviewers and KDIGO-assigned reviewers.

**Discussion:**

Rationale behind the selection of adopted statements and tailoring of others to suit our local facilities’ expertise and disease profile was discussed in the text with reasons.

**Conclusion:**

The provided guidelines aim to optimize patient care and outcome and suggest research areas lacking validated research recommendations.

**Supplementary Information:**

The online version contains supplementary material available at 10.1186/s43054-022-00119-w.

## Introduction and background

Nephrotic syndrome in children is usually primary (85–90%) showing glomerular involvement without an identifiable cause as infection, drugs, malignancy, or autoimmune disease. Evaluation might reveal an underlying systemic illness in 5–10% of patients. Most common histopathology is minimal change disease [1]. Most nephrotic children have steroid-sensitive nephrotic syndrome (SSNS) with MCD pathology, while 20% are steroid-resistant (SRNS), depending on the geographic area [2]. SRNS children show mostly focal segmental glomerulosclerosis (FSGS), minimal change disease (MCD), mesangial proliferative glomerulonephritis, and rarely membranoproliferative (MP) or membranous (MN) pathology [3]. SSNS outcome is satisfactory, 50% show frequent relapses or steroid dependence, and 3–10% show late steroid resistance [3]. A kidney biopsy is performed at disease onset only in children with atypical features and in all children with steroid resistance. Repeated kidney biopsy is indicated when prolonged (> 2 to 3 years) exposure to CNIs or in children with secondary steroid resistance. Egyptian children with idiopathic NS justified for biopsy showed MCD in 32.2%, FSGS in 45.2%, mesangio-proliferative in 9.7%, MP in 9.7%, and membranous in 3.2%. Indications for biopsy were SRNS in 79.5%, positive family history in 12.8%, age < 1 year in 2.5% or > 10 y in 10.3%, hypertension in 5.1%, and FR in 2.5% [4]. Genetic testing is not recommended in first presentation of the disease unless positive family history or syndromic or < 1y age. While it is of little value in SSNS, it is crucial in SRNS. IPNA clinical practice recommendations 2020, and KDIGO 2021 guidelines recommended routine evaluation of genetic mutations in children with SRNS if available [5–10]. Comorbidities of Idiopathic SSNS as infection, thrombosis, drug side effects are less marked than that observed in SRNS. The mainstay of treatment for IPNS is corticosteroids. Most children respond well to steroids within 4 weeks (SSNS); those who do not respond will be defined as SRNS patients at six weeks. Kidney outcomes In SSNS remain excellent, with < 5% risk of progression to chronic kidney diseases at 10 years after diagnosis [11]. In contrast, SRNS increased risk of progression to (ESRD) [5]. SSNS prognosis is correlated with morbidity of prolonged exposure to corticosteroids and steroid-sparing agents prescribed in frequently-relapsing or steroid-dependent disease. Idiopathic SSNS disease has a chronic, relapsing–remitting course, which tends to resolve spontaneously following puberty. However, in 15% to 25% of cases, it may progress to adulthood, maintaining the peculiar rapid response to corticosteroids in relapse. Small percentage of children may, become secondarily steroid-resistant with high chance both of progressing to kidney failure and to relapse after transplantation.

This CPG adaptation project for nephrotic syndrome is part of a national CPG program by the Egyptian Pediatric Clinical Practice Guidelines Committee (EPG), which was formulated in June 2018 by an initiative in collaboration with the faculty staff of 15 Egyptian universities' pediatrics departments and a national research center [12]. EPG was later affiliated with the Supreme Council of Egyptian University Hospitals, with the goal of defining the topics of pediatric evidence-based CPGs, assigning authors to them, and assisting in their adaptation in accordance with a national strategic plan (http://epg.edu.eg) [12]. The EPG follows the “Adapted ADAPTE” as a formal CPG adaptation method [13].

## Methodology

### Methods

**Fig. 1:**
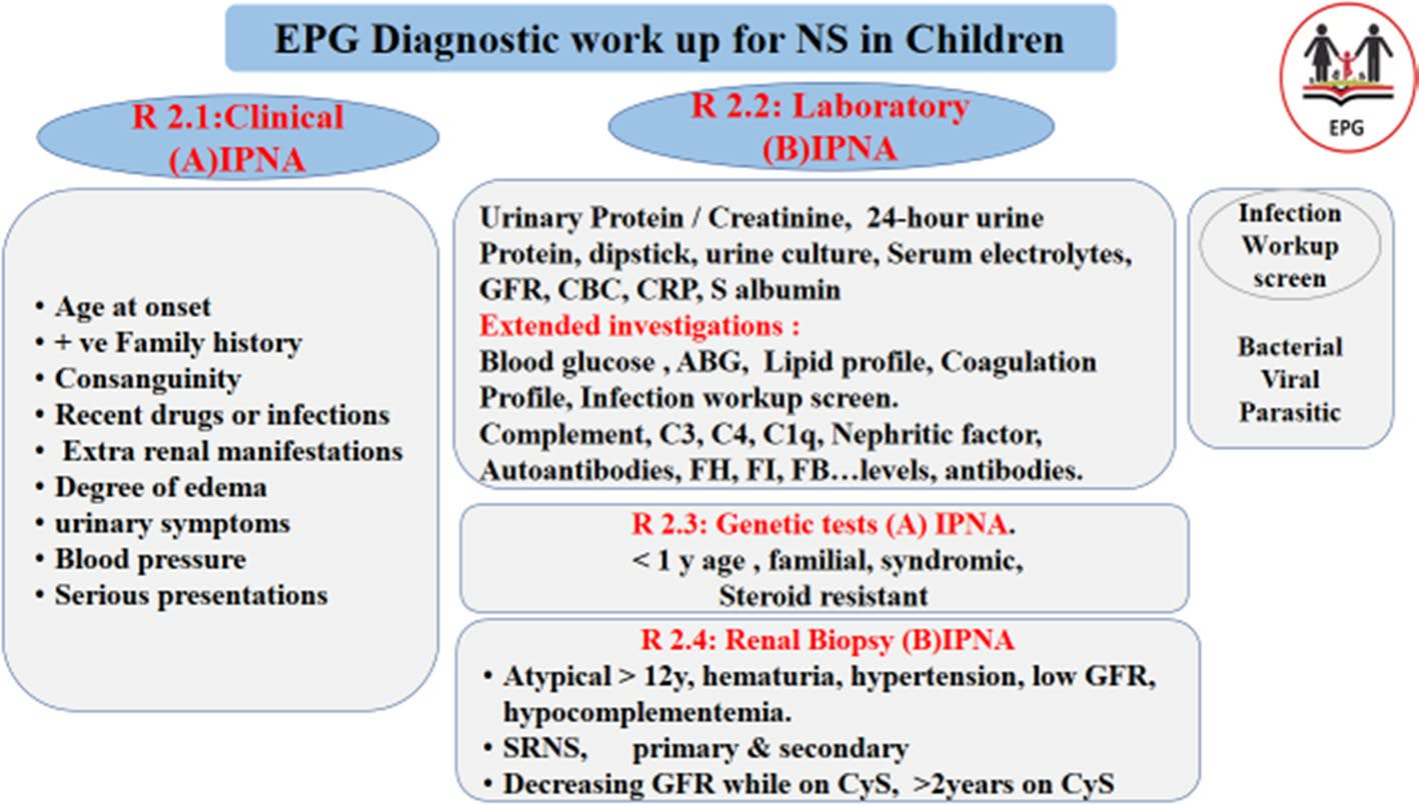
Diagnosis workup for nephrotic syndrome in children

**Fig. 2:**
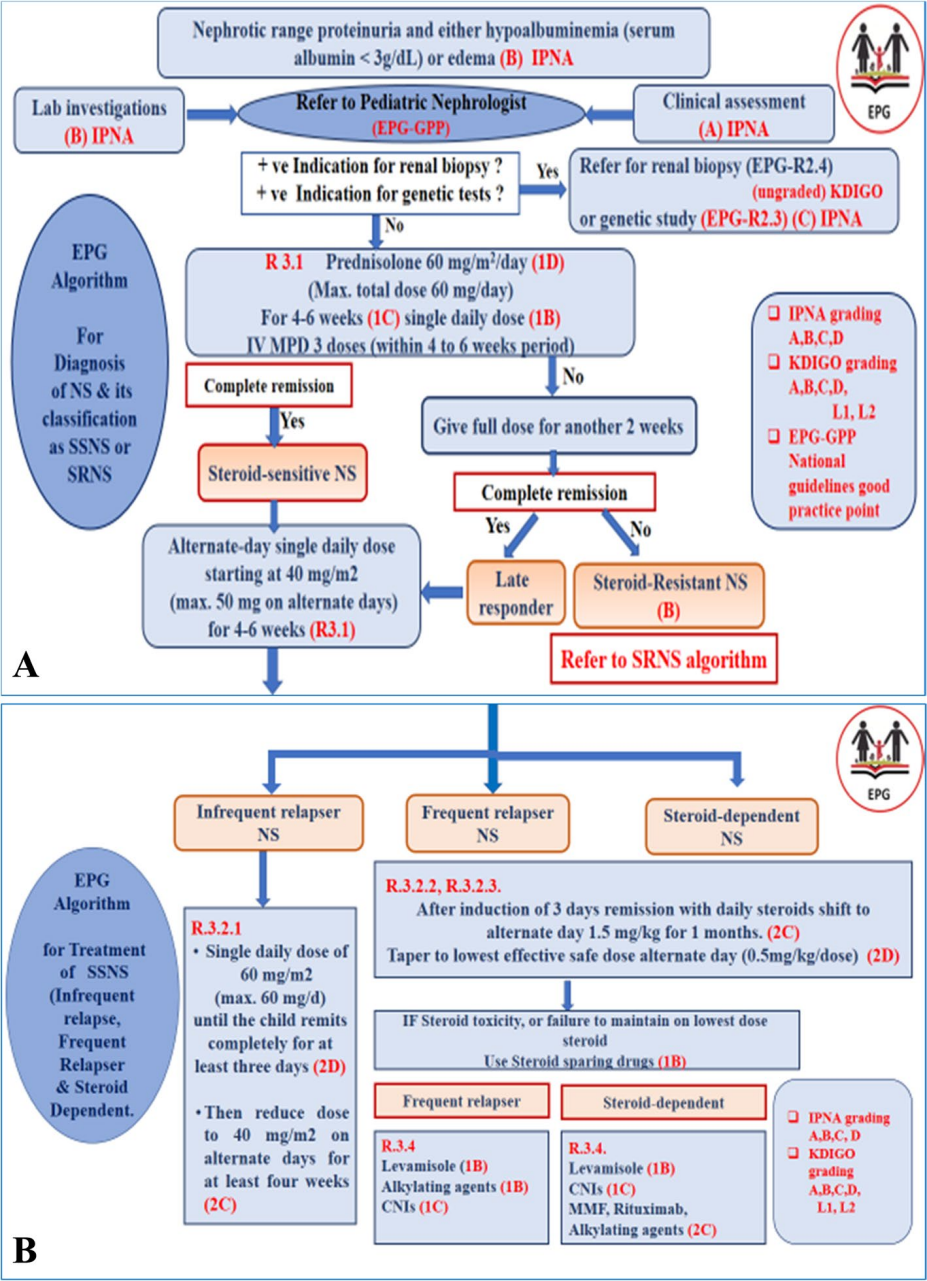
**A** EPG algorithm for diagnosis of NS and its classification as SSNS or SRNS. **B** EPG algorithm for treatment of SSNS (infrequent relapse, frequent relapser, and steroid dependent)

We followed the ‘Adapted ADAPTE’ CPG formal adaptation methodology that consists of three phases and 24 steps and tools [13–18] (Figs. 1, 2, 3, 4, 5, 6, 7, 8, and 9 in the Appendix). It was registered on the Practice guideline Registration for transparency (short prepare) international guideline registry with a registration number IPGRP-2021 CN374 Link http://www.guidelines-registry.org/index).

### Set-up phase 1

Nephrotic syndrome (NS) was highlighted as one of the prioritized health topics for the EPG CPG adaption initiatives during phase 1 (set-up). A preliminary search was carried out to revise and choose from the available existing Evidence Based NS CPGs to be our reference source**.** With 28 members, the Nephrotic Syndrome Guideline Adaptation Group (NS-GAG) was established. The NS-GAG included pediatrics and pediatric nephrology faculty academics and consultants from ten Egyptian universities. Two members of the NS-GAG were involved in the development of the Adapted ADAPTE and had previous experience with CPG adaptation. The CPG methodologists provided capacity training for the NS-GAG pediatric and nephrology consultants on the Adapted ADAPTE from the start of the project. Continuous virtual meetings extending through 1 year starting at March 2020 were scheduled for interactive communications between working group members. Our scope was pediatric nephrotic syndrome including (1) SSNS and (2) SRNS/diagnosis and treatment recommendations.

The target patient population for this CPG project include infants elder than three months till children and adolescents up to 18 years of age, presenting with nephrotic syndrome in a primary, secondary, and tertiary healthcare setting like clinics, emergency rooms, dialysis, or transplant wards. Excluded population were infants with congenital NS presenting during the first three months of life. The target users include physicians (viz. pediatrics, primary healthcare, family medicine, and pediatric nephrology), nurses, and clinical pharmacists.

Work group was divided into two panels assigned to cover each type (1) SSNS and (2) SRNS, with continuous communication at monthly virtual meeting with attendance of all working groups’ members. For clarity we will report the adapted recommendations of the EPG CPG/NS in two separate guideline formats: (1) steroid-sensitive and (2) steroid-resistant NS. This manuscript article will be devoted to SSNS aiming for, Proper initial diagnosis by practitioners and pediatricians and referral to pediatric nephrologists for extended assessment, proper treatment of cases at relapses and remissions, prevention and management of complications, Patient follow-up, family orientation with home monitoring of proteinuria, vaccinations, diet, and activity recommendations.

### Adaptation phase 2 SSNS

In phase 2, we identified 12 health questions, using the PIPOH model, including 5 questions for diagnosis and 7 for treatment (in the Appendix). The PIPOH model included the target patient population (P), intervention(s) (I), professionals and clinical specialties (P), outcomes (O), and healthcare setting or context (H). The literature search was conducted using MEDLINE/PubMed and Google Scholar portals. Eligible Source CPGs were evaluated using the Appraisal of Guidelines for Research and Evaluation (AGREE II) Instrument. AGREE II is a valid and reliable instrument with 23 items organized into six domains and is considered the gold standard for quality assessment of CPGs [14, 15]. (Documents for appraisal of source CPGs, health questions, PIPOH Model are included in the Appendix) The first draft of the adapted CPG marks the last step of this phase. The RIGHT- Ad@pt checklist, reporting the adapted Evidence-Based Clinical Practice Guideline for SSNS was used (see Appendix table).

### Finalization phase 3 SSNS

Involved in finalizing the initial draft of the adapted CPG, as well as determining whether it was acceptable and suitable to the Egyptian healthcare system. Thereafter, the document was sent out to a panel of four clinical (including one assigned from KDIGO) and one methodology external reviewer. Reviewers’ comments were revised. Updated draft was further reviewed within the NS-GAG, considering the national context. The finalized version of the revised CPG contained useful tools and strategies for implementation.

## Recommendations [19–30]

### Steriod sensitive nephrotic syndrome SSNS

**R 1: Definitions**

**WHAT are the Common Definitions Related to Nephrotic Syndrome?**

Definitions are summarized in IPNA 2020 and KDIGO 2021 (Table 1: refer to the Appendix).

**R 2: Diagnosis.** (EPG: Figs. 1 and 2A) **How to make a proper diagnosis of NS at its first episode?**

EPG recommends the following three practice points and R2 (1–5) to make a proper diagnosis for NS in children:

**R 2: (EPG-GPP 1):** At Primary health care settings, we recommend for **primary diagnosis** of NS:

**Clinical assessment** of oedema, blood pressure, urinary symptoms. Good history taking about: patient age at onset of disease, family history of similar complaints, previous infections, recent drugs, systemic disease).

**Basic laboratory assessment**: Urine analysis for sediments (hematuria, proteinuria), Protein/creatinine ratio, and urine culture, GFR, serum albumin, s. LDL-Cholesterol, complete blood count. **Nephrotic range proteinuria with low s albumin with and without oedema make a primary diagnosis of NS** (Table 2 IPNA 2020 and KDIGO 2021: refer to the Appendix).

**R 2: (EPG-GPP. 2)** We recommend **referral** of children with the primary diagnosis of NS to pediatric nephrologist, for proper extended diagnosis &management.

**R 2: (EPG-GPP. 3)** We recommend for pediatric nephrologists to **extend their assessment** as recommended by IPNA 20 to classify a nephrotic patient as steroid sensitive or steroid resistant, as each type will follow a separate guidance addressed to specific end users.

SSNS Guidance is addressed to practitioners, pediatricians, and pediatric nephrologists. Whereas SRNS guidance will be addressed to pediatric nephrologists.

**R 2.1:** We recommend for **extended Clinical assessment to** follow Table 2 (refer to the Appendix) **IPNA 2020** and **EPG flow-chart** (Fig. 1), focusing on; age at onset of disease, consanguinity, positive family history of kidney disease, extra renal manifestations, disease severity, degree of oedema, hypertension.

**R 2.2:** We recommend for **extended laboratory assessment** in atypical cases to follow **EPG flowchart** (Fig. 1) **and Table 2 IPNA 2020** (refer to the Appendix) focusing on:

- C3, C4, Anti ds DNA, ASOT, APLR, ANCA… **(B).**
- Infection screen (blood, urine culture, tuberculin test ©viral serology; HBsAg, HC, CMV, Epstein Bar, HIV, COVID Sars-2 antibodies **(C).**
- Imaging: abdominal and renal US, X-ray chest.

**R 2.3: Genetic tests for selected cases**

**WHEN to ask for GENETIC TESTING in first episode?**

**R 2.3.1: EPG-GPP**: We recommend **Early genetic testing** not waiting for 4 weeks steroid response **If:** Familial **(C)**, Syndromic **(C)**, < 1 year age of onset **(C)**, extra renal involvement suggesting hereditary disease **(C).**

**R 2.3.2: We recommend genetic testing after 4 weeks** of start of oral prednisone, for **all steroid resistant** if possible (**B) IPNA** or our high priority target groups (SR/FSGS or DMS, CNI resistant…. Refer to SRNS CPG to identify our target group).

IPNA 2020 recommends GT for infantile, familial syndromic and all SR, as early as possible in the transitional period (4–6 weeks) after steroid use with no response and before biopsy.

**KDIGO 2021** also recommended GT in SRNS when congenital and infantile (< 1 year of age), with syndromic features, familial **KDIGO 2021** (Fig. 43 Ps 152).

**R 2.4: Renal biopsy for selected cases WHEN to do KIDNEY BIOPSY in the first episode?**

**Rationale:** Since the prognosis for childhood NS is best predicted by patient response to steroid therapy &frequency of relapses in the first year after treatment, Therefore **KDIGO 2021** recommend renal biopsy at first episode only for those with atypical presentation or steroid resistance **PP: 4.2.1**. **KDIGO 2021**.

**R2.4.1: EPG-GPP** We recommend Early biopsy for **atypical** cases and not to wait for 4 weeks steroid therapy transitional period if:

- There is high index of suspicion for a different underlying pathology as macroscopic haematuria, hypertension, hypocomplementemia, etc.
- Children presenting with NS at age of > 12 years with hypertension and or haematuria.
- Early onset or rapid decline in GFR is not related to hypovolemia.

**2.4.2: EPG-GPP We recommend late biopsy as IPNA &KDIGO recommend for:**

- **Steroid resistant** NS (4 weeks after steroid therapy).
- Late failure to respond after initial response to steroids (**secondary SR).**
- Decreasing kidney function in children while **receiving CNIs** or those with prolonged exposure (2–3 years).

**R 2.5: WHO should manage nephrotic children? Referral Red Flags?**

**R 2.5.A** (**EPG-GPP):** EPG Work group panel recommend early referral of children with nephrotic syndrome to Pediatric Nephrologist at its first episode once diagnosed. Since Practitioners or pediatricians are commonly who make the primary diagnosis of Nephrotic syndrome; therefore, they should be informed about all criteria of referral. Early referral (upon diagnosis at its first episode) is better than late emergency referral of critical cases. Early referral helps in early classification as SSNS or SRNS, proper treatment of each type as well as management of complications cases by pediatric nephrologists.

Red flags justifying referral:

**R 2.5.B (EPG-GPP) Pre-treatment indications:**

- Age < 1 year, > 12 years, familial clustering of similar cases, extrarenal features
- Macroscopic hematuria, Hypertension, complement consumption, renal impairment,
- Hypo-complementemia, high antibody titer (ANCA, ADNA, APL),
- Positive viral serology (hepatitis B SA, hepatitis, or HIV antibodies), (+ tuberculin test + Blood culture in congenital or rheumatic cardiac patients or hydrocephalous with shunts).

**R 2.5.C: (EPG-GPP)** Post-treatment indications of cases difficult to manage:

- Failure to achieve partial or complete remission after 6-week standard dose steroids (primary SRNS).
- Secondary SRNS after initial response.
- Frequently relapsing or steroid dependent.
- Uncontrolled hypertension, infection, thrombosis.
- Steroid toxicity-renal impairment.

**R 3 (1–6): Treatment of Initial Episode of NS?** (Fig. 2A).

**R 3.1:** We suggest adopting **KIDIGO 2021** recommendations as oral corticosteroids to be given for 8 weeks (4-week daily steroids followed with 4 weeks at alternate day) OR 6 weeks daily followed with 6 weeks at alternate day **(1B)**. The standard dosing regimen for the initial treatment of NS is oral prednisone or prednisolone 60 mg/m^2^/day or 2 mg/kg/day (max 60 mg/day) for 4 weeks followed by alternate day 1.5 mg/kg/alternate day or 40 mg/m^2^ (maximum 50 mg/alternate day) for other 4 weeks or prednisone or prednisolone 6o mg/m^2^/day maximum 60 mg/day for 6 weeks followed with alternate day regimen 40 mg/m^2^/day max. 50 mg/d for other 6 weeks **KDIGO 2021 PP: 4.3.1.1**.

**We add the following local practice points:**

**R 3.1.A: EPG-GPP** divided dose is accepted in children with gastric upset since single dose is preferred for better adherence and not superiority. **(KDIGO 2021).**

**R 3.1.B: EPG-GPP** although dose calculation per surface area is more accurate in children. Per kg calculation may be accepted for simplification provided no under dosing [24–27].

**R 3.1.C: EPG-GPP** 12-week total duration for steroids may be prolonged in late responders **(KDIGO 2021 draft),** therefore total steroid duration remains as open point of research.

**R 3.2: HOW to maintain long remission on recurrence for:**

**Infrequently relapsing? Frequently relapsing, steroid dependent?**

**R 3.2** The initial approach for induction should include prednisone as a single daily dose of 60 mg/m2 or 2 mg/kg (maximum 60 mg/day) until the child remits completely for at least three days. **KDIGO 21 PP: 4.3.2.1**. Then:

**R 3.2.1**: Steroid maintenance for **infrequently relapsing:** after achieving complete 3 days remission with single daily dose of prednisone 60 mg/m^2^ or 2 mg/kg (maximum of 60 mg/day) **KDIGO 21 PP:4.3.2.2**. Children are suggested to have alternate days (40 mg/m^2^ per dose or 1.5 mg/kg per dose (maximum 50 mg/day) for at least 4 weeks. **(2C) KDIGO2021.**

**R 3.2.2. A: Frequently relapsing** SSNS Children without steroid toxicity are suggested to be treated with the same glucocorticoid regimen in subsequent relapses. Prednisone is suggested to be given on alternate days in the lowest dose (Optimal dose ≤ 0.5 mg/kg) to maintain remission without major adverse effects PP**: 4.3.2.4**. **KDIGO2021.**

**R 3.2.2. B: EPG-GPP does not accept daily low-dose** steroids on failure of alternate day low dose unless total weekly dose is kept the same but divided as daily dose. **KDIGO 2021** suggests daily prednisone at the lowest dose to maintain remission in children without major adverse effects in FR/SSNS when alternate-day prednisone therapy is not effective **(2D).**

**R 3.2.3: EPG-GPP Steroid Dependent SSNS children:** We accept low-dose steroids for low dose–dependent patients (alternate day dose ≤ 0.5 mg/kg) provided; no steroid toxicity, continuous patient monitoring with dose titration, patient preference and accept of potential harm **(KDIGO 2012)** [28]. **KDIGO2021** does not recommend low-dose steroids for SD as they recommend steroid-sparing drugs for all SD to avoid steroid toxicity. **KDIGO 2021 (1B) PP 4.3.2.2.**

**R 3.3:** We recommend for frequently relapsing or steroid dependents children who are currently on alternate-day prednisone or off glucocorticoids **during episodes of the upper respiratory tract** and other infections, daily prednisone (0.5 mg/kg) for 5 to 7 days to reduce the risk for relapse. **(1C).**

**R 3.4: WHICH STEROID SPAIRING DRUGS to recommend for FR and SD?**

**Rationale:**

It has been well accepted that corticosteroid-sparing agents were recommended to be prescribed for children with (FR) SSNS and (SD) SSNS, who develop steroid-related adverse effects. **(1B) KDIGO 2012.**

**KDIGO 2021** Suggests low dose steroids (optimally alternate day dose ≤ 0.5 mg/kg) as maintenance only for **FR who respond to** the low glucocorticoid dose without serious toxicity **PP: 4.3.2.4. (B) KDIGO2021**. Whereas all **SD should use** steroid-sparing drugs to avoid long-term use of steroids. (**1B) KDIGO 2021**, PP **4.3.2.2**

- Patients should be ideally in remission with glucocorticoids prior to initiation of steroid-sparing drugs. Coadministration of glucocorticoids is recommended for 2 w following initiation of steroid-sparing drugs **PP 4.3.2.5. KDIGO 2021**
- Choosing the most appropriate drug is related to patient resources, adherence, tolerance, adverse effects, contraindications, and drug availability. **PP: 4.3.2.6. KDIGO 2021**
- For **FR**, levamisole and oral cyclophosphamides are preferred. For **SD** MMF**, rituximab**, cyclosporine, and to a lesser extent cyclophosphamides are suggested **PP: 4.3.2.6. (1B). KDIGO 2021.** Dose, duration, efficacy, and complications for each are summarized in Table 3 (refer to the Appendix. IPNA 2020 P 1541).

**R 3.4: We suggest for frequently Relapsing** (Fig. 2B) **EPG-GPP**, in case of resistance to low and safe steroid dose, to use levamisole as our first choice, to be replaced with cyclophosphamides considering its cumulative dose, or azathioprine. **(1B KDIGO 2021).**

**We suggest in steroid-dependent EPG-GPP**, CNIs as our second choice after levamisole in children. Rituximab is an expensive drug, not covered by medical insurance and its use looks risky in countries endemic to hepatitis and other infectious diseases **(2C).** Our limited experience in the use of Mizorbine unlike adults also restricts its common use in children **(EPG-GPP).**

**R3.5: Management of complications (oedema, infections)**

**R. 3.5.1: EDEMA**

**Rationale**

**Patients with mild oedema** do not require diuretic therapy. Corticosteroid therapy for relapse results in diuresis within 1 week, enabling loss of retained extracellular fluid. Patients are advised to limit sodium intake.

**For patients with moderate oedema without hypovolemia**

- We recommend oral furosemide as first-line therapy (2–4 mg/kg/day**) (B) Japanese 2014**
- We suggest additional use of hydrochlorothiazide (2–4 mg/kg/day) or metolazone (0.1–0.2 mg/kg q12–24 h) to augment diuresis with Monitoring for hypovolemia, hypokalemia **(B) Japanese**. Spironolactone has limited diuretic efficacy but a potassium-sparing agent in patients receiving high-dose furosemide. Use of amiloride is not advised. EPG-GPP [20]
- We suggest that patients with furosemide-refractory oedema be managed as follows: (i) combination of loop diuretics with thiazide and (ii) co-administration of human albumin with IV furosemide. **(X) IPNA2020.** Unresponsive to oral furosemides due to gut oedema indicates switching to IV therapy as 1–2 mg/kg q 8–12 h.
- **Patients with severe oedema.** We recommend loop diuretics furosemide unless hypovolemic to avoid thrombosis and AKI. **(X) IPNA 2020**. Hypovolemia in NS results after vomiting, diarrhea, and diuresis.
- We suggest treating patients with severe and refractory oedema with hypovolemia, human albumin infusion **(C) IPNA 2020.** Starting dose 20–25% albumin, 0.5–1 g/kg IV over 4–8 h, adding furosemide 1–2 mg/kg iv in the middle and at the end of infusion **(C) IPNA 2020.** Blood pressure and heart rate monitoring with slowing infusion with any sign of overload. **(X) IPNA 2020.**

**R 3.5.2: Infection**

- We suggest that serious bacterial infections associated with nephrotic syndrome be managed as indicated**. (X) IPNA2020. Referral to pediatric nephrologists is crucial. EPG-GPP**
- Follow-up to prevent Infection with infection control measures and vaccinations.
- We suggest immune globulins for children with recurrent infections or low serum IgG levels (**D) IPNA2020.**
- We do not recommend routine antibiotics.** (C) IPNA2020** We suggest cotrimoxazole in patients on rituximab (5–10 mg/kg/day 3 times weekly 3–6 m) **(C) IPNA2020** We recommend receiving all **vaccinations** as recommended below.

**Rationale:**

Infections are the chief complication in patients with SSNS, accounting for most hospitalizations. Contributing factors include the use of immunosuppressive agents, anasarca, and urinary losses of IgG. **Peritonitis** is the most common severe infection, followed by **pneumonia** and **cellulitis** [26]. The diagnosis and treatment of severe infections should follow standard guidelines. Apart from vaccines, there is no evidence of routine antibiotics **(C) IPNA 2020.**

**Viral infections** several viruses, including rhinovirus, adenovirus, influenza, parainfluenza, enterovirus, and respiratory syncytial and Epstein–Barr viruses, might trigger disease relapses. varicella, zoster, and influenza might cause serious morbidities **KDIGO 2012/2021**. Infections such as severe acute respiratory syndrome coronavirus 2 infection with severe acute respiratory syndrome coronavirus 2 **(SARS COVID 2),** the etiological agent of coronavirus disease (COVID-19) poses challenges in the management of patients with nephrotic syndrome [29, 30]. While children show mild disease, patients on immunosuppression constitute a high-risk group that is predisposed to adverse outcomes. Affected patients are at risk of AKI, particularly if associated with hypovolemia or aggressive use of diuretics [29]. Most expert groups advise reduction of immunosuppression or steroids to acceptable levels, limiting the use of biological agents, balancing the risk of disease relapse against infection.

**R 3.5.3: Prevention of thrombosis**

We recommend mobilization and avoiding central lines except for specific and transient need **(X)** strong recommendation. Insufficient evidence for routine anticoagulant with no previous history or risk of thrombosis (not graded). We suggest low molecular weight heparin in those patients with: *previous history of thrombosis, central lines, hereditary thrombophilia predisposition, infection or dehydration **(C)**. We suggest thrombophilia screen for protein C, S, Anti thrombin, factor V genes in those with a positive family history of thrombophilic predisposition **(C) IPNA 2020.**

**R: 4.1: How to follow your patient?**

- Diet: We recommend fat restricted diet **(C1) Japanese 2014** balanced fluids uptake **(C) IPNA 2020**, salt moderation **(C) IPNA 2020**, and moderate exercise **(C2) Japanese 2014.**
- Family orientation with relapsing course of the disease, how to use uro-strips, adherence to steroid therapy and monitoring for its side effects.
- Blood pressure assessment and control.
- Laboratory testing to follow proteinuria, GFR, lipids profile, urine & blood glucose.
- Infection screen and drug monitoring for those under immunosuppressives.
- Vaccinations refer to **(R 4.2) (B1) Japanese 2014, IPNA 2020 (A).**
- Growth follow-up.
- Vitamin D and calcium supplements, pump inhibitors **(C) IPNA 2020, KDIGO 2021 (Fig. 43 P.s152).**
- In patients with SSNS and normal vitamin D levels, supplementation is not required. However, in FRNS or SDNS children with a known vitamin D deficiency, a reduction of bone mineral content can be prevented by oral supplementation of calcium and vitamin D.
- **KDIGO 2021** reported absence of sufficient evidence to recommend prophylactic use of proton-pump inhibitors in children with NS in absence of risk factors as gastric symptoms.
- Management of complications (infection, thrombosis, steroid toxicity, immunosuppressive side effects) with immediate referral to Pediatric Nephrologists for urgent interference.** (EPG-GPP).**

**R 4.2: IMMUNIZATIONS IN CHILDREN WITH SSNS**

**R 4.2:** To reduce the risk of serious infections in children with SSNS, **IPNA 2020** suggest reviewing the **child vaccination status** at disease onset **completing all vaccinations without delay** especially for encapsulated bacteria (pneumococcal, meningococcal, Hemophilus influenza) and if possible, varicella-zoster virus

- Give **pneumococcal**, meningococcal, and varicella vaccination to the children **(A)IPNA 2020.**
- Give **influenza** vaccination annually to the children and their household contacts **(A)IPNA 2020.**
- Live vaccines are contraindicated in children receiving corticosteroid-sparing immunosuppressive agents **(X) Strong recommendations IPNA 2020.**
- Immunize healthy household contacts with live vaccines to minimize the risk of transfer of infection to the immunosuppressed child but avoid direct exposure of the child to gastrointestinal, urinary, or respiratory secretions of vaccinated contacts for 3–6 weeks after vaccination.
- Following **close contact with varicella infection**, give nonimmune children on immunosuppressive agents, varicella zoster **immune globulin**, if available **(A**). Treatment with **acyclovir** 10 mg/kg/7 days **(not graded), varicella vaccine in remission (C) IPNA 2020**.

## Discussion [31–37]

**This CPG adaptation project** for Nephrotic Syndrome is part of a national CPG program by the Egyptian Pediatric Clinical Practice Guidelines Committee (EPG), with the goal of defining the topics of pediatric evidence-based CPGs, assigning authors to them, and assisting in their adaptation in accordance with a national context. The EPG follows the 'Adapted ADAPTE' as a formal CPG adaptation method.

**EPG Recommendations** for nephrotic syndrome in children **1—SSNS/Diagnosis, treatment, are mostly** adopted for use from both **IPNA 2020 and KDIGO 2021** after permission. Few were modified and tailored to suit our community.

### [I] Adapted recommendations diagnosis workup modification:

**(EPG) adopted IPNA 2020** recommendations for clinical, laboratory and imaging assessment (basal and extended) for infants or children with suspected diagnosis of nephrotic syndrome. However, Panel added these local good practice points for **customization** to our local text, (Fig. 1A, B)

**R 2: Early referral** to **pediatric nephrologist (PN) upon primary diagnosis of** NS at its first episode is recommended. Primary diagnosis is **based on** nephrotic range proteinuria > 1 g/m^2^/day or Upc > 200 mg/mmol morning sample or 3 + dip sticks, serum albumin ˂ 3 mg/dcl with or without oedema. Further assessment by PN to identify hereditary or secondary from idiopathic types and to classify patients as steroid responsive or resistant assure better patient care **[EPG-GPP 1.2.3] [R 2. 1–5].**

The fact that some families insist to keep their children under their pediatrician care justify practice points defining **high-priority group for early referral before starting steroids:** (1) with early onset disease <1 year, family cluster for similar cases, syndromic features, or atypical presentations **[R 2.5.B] and** (2) **for cases difficult to manage as primary and secondary SR, FR, SD, critical and complicated cases [R 2.5.C].**

**R 2.2: EPG-GPP Early Extended lab immunology work is recommended in atypical cases, with a high suspension of being secondary to infection or autoimmune disease or of non-MCD pathology**, rather than to be delayed to transitional period after 4 w of standard steroid dose. This should include (ANA, ADNA, APL ANCA), as well as an **infection screen** for all patients with high index suspension (tuberculin test, viral serology HBV, HCV, CMV, HIV, SARS CoV-2).

**Our reasons:**

1. Being a country with endemic foci for some infectious diseases as hepatitis, tuberculosis, and schistosomiasis, national EPG should focus on diagnosis, prevention, and treatment of infection-related NS before starting steroids or other immune suppressive drugs if indicated (Fig. 1, diagnosis workup).
2. Exclusion of secondary types as autoimmune disease SLE, ANCA, IgA, and malignancy. Complement level and profile (C3, C4, C1q) is crucial might be helpful for differential diagnosis of C3/DDD

**R 2.3: EPG-GPP Early Genetic testing** (before starting steroids, and not to wait for 4 weeks steroid response) for cases < **1 year age at disease, onset, family clustering, syndromic** features with extrarenal manifestations (Fig. 1) **(R 2.3).**

All SR cases are indicated for GT by IPNA if possible. **EPG for SR [part 2]** framed our target priority group of SR for GT. Currently, there is no diagnostic or prognostic utility for biomarkers of MCD in SSNS although identified variants as risk factors were revealed in few studies [26, 31–33].

**R 2.4: EPG-GPP** Early kidney biopsy (and not to wait for 4 weeks steroid response) for cases with atypical presentation (> 12 years age (Fig. 1)) **R 2.4.** infants, presence of extra-renal manifestations, hematuria, hypertension, and hypocomplementemia impaired GFR (Fig. 1) **[R 2.4]**

Late kidney biopsy for primary **steroid resistance** after 4 weeks of therapy or secondary SRNS following initial response or patients on **cyclosporine therapy with decreasing GFR** are recommended as **adopted** statements for **IPNA 2020 and KDIGO 2021**.

**R 2.5: EPG-GPP** Since **referral** recommendations to pediatric nephrologists are crucial for atypical cases and PNCs are the well-equipped areas for proper management: We recommend sustained support of these PNC centers with all needed facilities: (genetic testing, laboratory tests for immunology, infection screen, drug monitoring, renal biopsy, immunosuppressive drugs, with medical insurance cover). (**R 2.1, R 2.5).**

### [II] Adapted recommendations treatment adaptation

**R 3.1: Steroids (first episode)**

**EPG adopt KIDIGO 2021** first episode R as 4–6 weeks daily steroids 2mg/kg followed with 1.5 mg/kg alternate day dose for other 4–6 weeks **(R 3.1).**

**EPG Panel added these practice points:**

**R 3.1: EPG-GPP** Since **single dose** assure adherence in children and not superiority **(KDIGO 2021),** children with gastric upset not tolerating high dose can use a divided dose. Although **dose calculation** per surface area is more accurate in children [24, 25, 31], however, calculation per kg is easier but under-dosing is risky [34, 35]. Although the **total duration** for steroids was adopted as 12 weeks by **KDIGO 2021**, prolongation for late responders remains a future research area [31, 36].

**R 3.2 Steroids for infrequently relapsing**

**R 3.2: KDIGO 2021 adopted** as daily steroids 2mg/kg till 3-day remission followed with 1.5mg/kg alternate day dose for other 4 weeks.

**R 3.2.1: Steroids for (FR) and R 3.3: (SD)**

**EPG adapted KDIGO 2021** recommendation for low-dose steroid use (optima dose 0.5mg/kg at alt day) to maintain long remission in FR or SD by adding the following practice points **(R 3.3)**

**R 3.2 1: EPG-GPP** Suggest its use after induction of remission in FR if: no steroid toxicity, steroid, an effective dose that maintains long remission without side effects **is < 0.5 mg/kg**, with continuous patient monitoring, based on dose titration.

**R 3.3:** EPG-GPP: For **SD** low-dose steroids use versus steroid-sparing drugs should be based on effective dose, patient preference, potential harm, and drug availability [28] **[KDIGO 2012], **[26]** [ASIAN 2021].**

**R 3.3.B:** EPG-GPP **daily** low dose 0-5mg/kg is **not accepted** for FR or SD, although accepted by **[KDIGO 2021]** for FR showing no toxicity. However, we accept the total weekly alternate day dose to be divided as daily over the 7 days (0-25 mg/kg daily) to maintain daily satisfactory blood level for corticosteroids **EPG-GPP** [37].

**R3.4: Steroid-sparing drugs locally justified for FR and SD: R 3.5** (Fig. 2B)

**In Egypt**, in a cohort study including 130 patients recruited over 2 years as SRNS (51 cases), FR and SD (79 cases). Drug efficacy to keep long remission in FR and SD was higher with cyclophosphamides (85.7%), CsA (83.3%), as compared to MMF (50%), levamisole (55%) [4]. Because of its low costs and minimal side effects, levamisole is preferred as the first option therapy for FR/SD in Egypt. It was used in 50.6% of recruited FR/SD cases, whereas cyclophosphamides (35.4%), Aza (12.7%), cyclosporine (7.6%), and MMF (2.5%) [4].

**CANADIAN SOCIETY** of Nephrology Commentary on **KDIGO 2012** Supported Canadian use of cyclophosphamides **(1B)** being commonly used in FR and SD in Canada whereas chlorambucil and levamisole are not available. They also considered **KDIGO 2012** equal grading for both cyclophosphamides and cyclosporine for SD, thus raising the debate of 3 months cyclophosphamides versus 1 year cyclosporine. For frequent relapsers, cyclophosphamides **(1B)** were better graded than cyclosporine **(2C).**

**R 3.4. EPG-GPP We suggest levamisole** as our first option drug for FR or SD being cheap, available, effective, less side effects [4, 27], which was also suggested in sub-Saharan Africa [27] -and **[KDIGO 2012–2021].**

**EPG-GPP for FR:** We suggest, on levamisole failure, to use **cyclophosphamides** or MMF or azathioprine guided by patient cumulative dose of cyclophosphamides, tolerance, and response to the drug [27, 28].

**EPG-GPP for SD:** We suggest, on levamisole failure, to use CSA or MMF considering that MMF was less effective than CSA in our Egyptian randomized control studies and that CsA nephrotoxicity and patient dependency justify its gradual substitution with MMF [4] to be considered after 2 years or whenever GFR drop **[KDIGO 2021]. Rituximab** is expensive drug not covered with insurance, considered only in children with SD and SSNS who are resistant to other steroid-sparing agents **(2C) KDIGO 2012. Exclusion of mizorbine** is related to our little experience in children as suggested not to be used in FR and SD **(2C) KDIGO 2012.**

## Conclusion

Adaptation guidelines are very helpful for countries with limited resources. The ADAPTE process is a comprehensive tool for the development of high-quality CPGs for healthcare institutions in developing countries. Our collaboration and adaptation of CPG produced by a relevant organization such as KDIGO or an international specialized society as IPNA aim to optimize patient care with the most beneficial and least harmful (evidence-based) interventions customized to our culture, considering patient values and references.

## Supplementary Information

**Additional file 1:** **Appendix.** EPG Methodology: **Fig 1.** Selection criteria for reference CPGs. **Fig 2.** Critical group appraisal of IPNA 2020 using the AGREE II Instrument. **Fig 3.** Critical group appraisal of JSPN 2014, using the AGREE II Instrument. **Fig 4.** Critical group appraisal of KDIGO 2012 & 2021 using the AGREE II Instrument. **Fig 5.** IPNA Evidence Grading. **Fig 6.** KDIGO Evidence Grading. **Fig 7.** Modified adapt tool 6 health questions (PIPOH) checklist. **Fig 8.** Development of recommendations. **Fig 9.** Adaptation steps. **Table A.** The RIGHT-Ad@pt checklist. RIGHT = Reporting Items for practice Guidelines in Health care. IPNA 2020 & KDIGO 2021 Tables. **Table 1.** Definitions related to Nephrotic Syndrome in Children. (IPNA 2020 and KDIGO 2021). **Table 2.** Initial workup and follow-up for a child with steroid-resistant nephrotic syndrome (IPNA 2020). **Table 3.** Steroid sparing therapy in SSNS (KDIGO 2021).

## Data Availability

Available on the Website of National Egyptian Guidelines after Publication.

## Abbreviations

ANA: Antinuclear antibodies
ANCA: Anti-neutrophil cytoplasmic antibody
Anti-ds DNA: Anti-double stranded-DNA
Anti-PLA2R: Anti-phospholipase A2 receptor antibody
ASOT: Anti-streptolysin O titer
C1q: Complement component C1q
C3: Complement 3
C4: Complement 4
CNIs: Calcineurin inhibitors
CPG: Clinical practice guideline
DMS: Diffuse mesangial sclerosis
EPG: Egyptian Clinical Practice Guideline Committee
EPG-GPP: Egyptian Clinical Practice Guideline Committee-Good Practice Point
EPG-WG: Egyptian Clinical Practice Guideline Committee-Work Group
ESRD: End stage renal disease
FR: Frequently relapsing
SD: Steroid dependent
CsA: Cyclosporine A
FSGS: Focal segmental glomerulosclerosis
GFR: Glomerular filtration rate
HBsAg: Hepatitis B virus surface antigen
HCV Ab: Hepatitis C virus antibodies
HIV: Human immune deficiency virus
IFR: Infrequently relapsing
MCD: Minimal change disease
MMF: Mycophenolate mofetil
NGL: National Egyptian CGLs Committee
NS: Nephrotic syndrome
NS-GAG: Nephrotic Syndrome Guideline Adaptation Group
RTX: Rituximab
SLE: Systemic lupus erythematosus
SRNS: Steroid resistant nephrotic syndrome
SSNS: Steroid sensitive nephrotic syndrome
