# Supplementary material for: Egyptian evidence -based pediatric clinical practice adapted guidelines for management of [1] steroid sensitive nephrotic syndrome (EPG/SSNS 2022)
Source: Egypt Pediatric Association Gaz. 2023 Feb 27;71(1):13. doi: 10.1186/s43054-022-00119-w (PMC9968631; doi:10.1186/s43054-022-00119-w)
Supplement: Supplementary file 1 — Additional file 1: Appendix. EPG Methodology: Fig 1. Selection criteria for reference CPGs. Fig 2. Critical group appraisal of IPNA 2020 using the AGREE II Instrument. Fig 3. Critical group appraisal of JSPN 2014, using the AGREE II Instrument. Fig 4. Critical group appraisal of KDIGO 2012 & 2021 using the AGREE II Instrument. Fig 5. IPNA Evidence Grading. Fig 6. KDIGO Evidence Grading. Fig 7. Modified adapt tool 6 health questions (PIPOH) checklist. Fig 8. Development of recommendations. Fig 9. Adaptation steps. Table A. The RIGHT-Ad@pt checklist. RIGHT = Reporting Items for practice Guidelines in Health care. IPNA 2020 & KDIGO 2021 Tables. Table 1. Definitions related to Nephrotic Syndrome in Children. (IPNA 2020 and KDIGO 2021). Table 2. Initial workup and follow-up for a child with steroid-resistant nephrotic syndrome (IPNA 2020). Table 3. Steroid sparing therapy in SSNS (KDIGO 2021). [file 43054_2022_119_MOESM1_ESM.docx]

| - **Egyptian Pediatric Association Gazette** **Electronic** **ISSN: 2090-9942 Springer**   **Journal of the** [**Egyptian Pediatric Association**](http://www.egyptpediatrics.org/) |
| --- |

**Egyptian Pediatric Clinical Practice Adapted Guidelines Evidence Based**

**[1] Steroid Sensitive Nephrotic Syndrome: SSNS 2022.**

**Appendix**

**Methodology Documents**

**1-Selection criteria for reference CPGs**

**
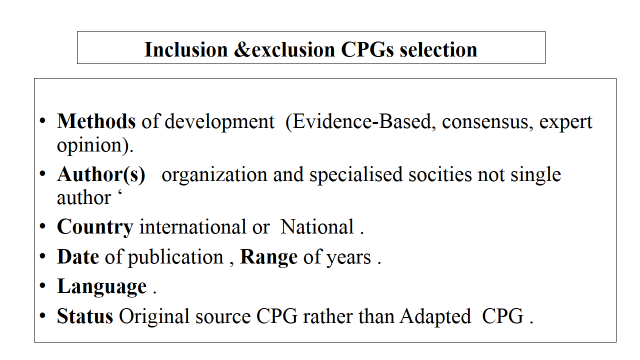
**

**(Fig 1 Methodology )**

**2- List of retrieved Guidelines**

1. IPNA clinical practice recommendations for the diagnosis and management of children with steroid-resistant nephrotic syndrome.

Available at: Pediatric Nephrology (2020) 35:1529–1561 <https://doi.org/10.1007/s00467-020-04519-1>.

1. Clinical practice guideline for pediatric idiopathic nephrotic syndrome 2013: medical therapy. Japanese Society of Nephrology and The Japanese Society for Pediatric Nephrology 2015.

Available at: Clin Exp Nephrol DOI 10.1007/s10157-014-1030-x.

1. KDIGO Clinical Practice Guideline on Glomerular Diseases

Available at: volume 2 | issue 2 | June 2012 <http://www.kidney-international.org>

**d-** Kidney Disease: Improving Global Outcomes (KDIGO) Glomerular Disease Work Group. KDIGO 2021.Clinical Practice Guideline for the management of Glomerular Diseases. KidneyInt. 2021; 100 (45):51-S276.

Available at <https://kidigo.org/guidelines/gd/(acessed29/09/2021)(KDIGO2021>

1. **Critical group appraisal of reference guidelines:**
2. IPNA clinical practice recommendations for the diagnosis and management of children

with steroid-resistant nephrotic syndrome, using the AGREE II Instrument.


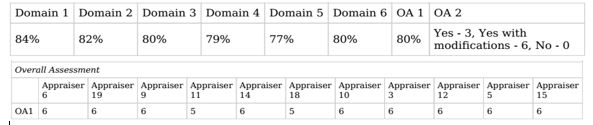


**(Fig 2 Methodology)**

1. Evidence-Based Clinical Practice Guidelines for Nephrotic Syndrome JSPN 2014,

using the AGREE II Instrument.

**
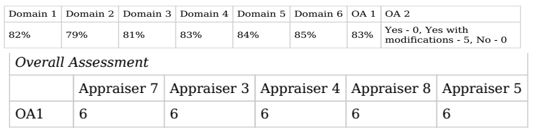
**

**(Fig 3 Methodology)**

1. KDIGO Clinical Practice Guideline on Glomerular Diseases, using the AGREE II Instrument.
   **
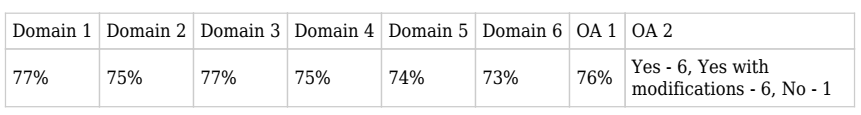
**

**(Fig 4 Methodology)**

**4. Evidence Gradings:**

1. **IPNA Evidence Grading**


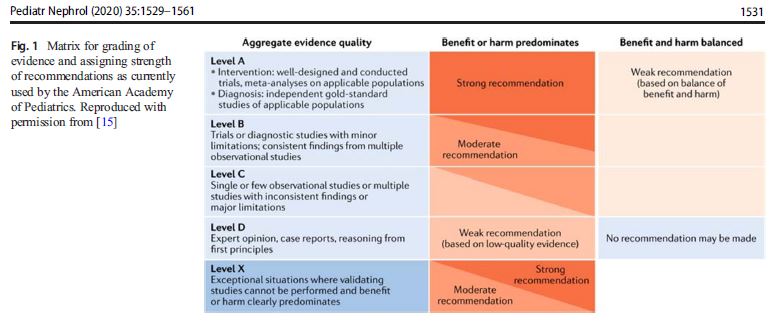


**(Fig 5 Methodology)**  **Reproduced with permission IPNA 2020.**

**B) KDIGO Evidence Grading**

**
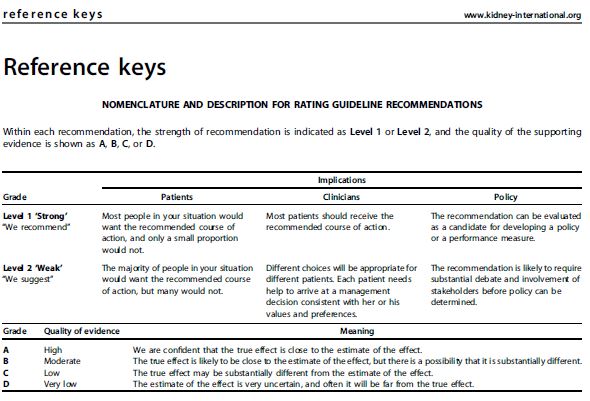
**

**(Fig 6 Methodology)**  **Reproduced with permission KDIGO 2021.**

1. **Health Clinical Questions – 5-(PIPOH) SSNS used to develop this Adapted CPGL.**

1- What are the Definitions related to Nephrotic Syndrome?

2- What are the recommended investigations for diagnosis?

3- What are the indications of renal biopsy in the initial presentation of NS?

1. Where should we manage infants, children and adolescents presented with first episode

of NS? When to refer to pediatric Nephrologist as complicated cases?

5- What is the first line treatment drug in first episode?

6- What are the recommended vaccinations?

7- What is your steroid maintenance protocol after remission in first episode and recurrence?

8- What are your (Diet, Fluids, Activity) recommendations?

9- What information & instructions you like to share with the family during follow-up?

10- Should vitamin D & Calcium supplements be routinely given to FR&SD?

11- What are the laboratory tests and how frequently needed for follow-up?

12- When should steroid sparing drugs be started in FR&SD?

13- What is the preferred steroid sparing drugs in FR&SD?

14- What are recommended doses & duration and monitoring of these drugs?

**6- PIPOH Adaptation Model**

• **P** (**Patients, target population):** children aged 1–12 years with non- congenital, SSNS

• **I** **(Interventions and practices considered/ guideline category):**

Life saving measures. /Pharmacological treatment. /Non- pharmacological treatment.

• **P (Professionals / intended or target users and clinical specialties):**

Primary health care physicians at Ministry of Health (MOH)/General practitioners/Family medicine specialists/Pediatricians /Nephrologists.

• **O** **(Major outcomes considered):** Primary Outcome: decrease the morbidity and mortality

Secondary Outcome: proper treatment

• **H** **(Healthcare settings) Primary** & secondary health care setting Outpatient clinic, emergency room.


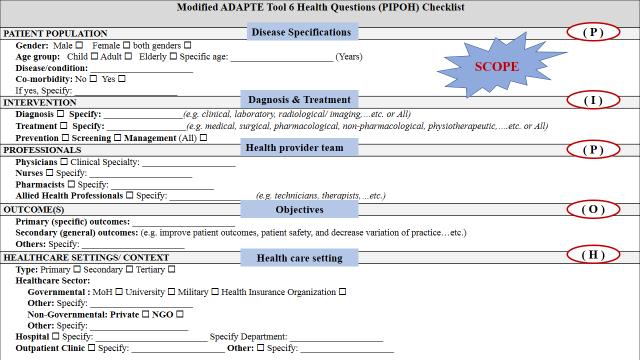


**(Fig 7 Methodology)**

**7-Development of Recommendations:**

Search and rating for EB answers, formulation of recommendations, editing, peer reviewing, validation, approval, implementation and dissemination, review/update.


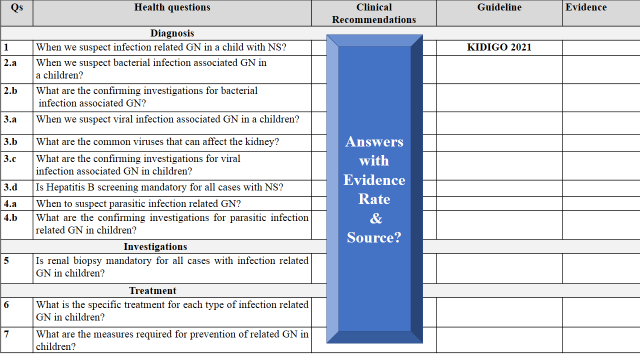


**(Fig 8 Methodology)**

**8-Adaptation steps**


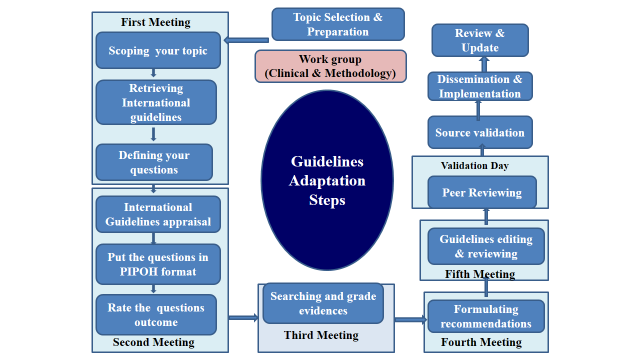


**(Fig 9 Methodology)**

**9- RESEARCH AND REPORTING METHODS**

**Reporting Tool for Adapted Guidelines in Health Care: RIGHT-Ad@pt Checklist**

**Table A Methodology : The RIGHT-Ad@pt checklist. RIGHT =**

**Reporting Items for practice Guidelines in Health care.**

| 7 sections, 27 topics and 34 items SSNS | | | | |
| --- | --- | --- | --- | --- |
|  | Basic information | Assessment | Pages | Notes |
| 1 | Identify the report as an adaptation of practice guideline(s), that is include "guideline adaptation", "adapting",  "adapted guideline/recommendation(s)", or  similar terminology in the title/subtitle. | - Yes - No - Unclear | 1 cover page  9 (Guideline Status)  74 (Methodology) |  |
| 2 | Describe the topic/focus/scope of the adapted guideline. | - Yes - No - Unclear | 1 Cover page 21 (Scope and purpose, PIPOH) |  |
|  | Cover/first page |  |  |  |
| 3 | Report the respective dates of publication and the literature search of the adapted guideline. | - Yes - No - Unclear | 1(Cover page)  5(Acknowledgment)  103 (Documents) |  |
| 4 | Describe the developer and country/region of the adapted guideline. | - Yes - No - Unclear | 1(Cover page)  5 (Acknowledgment)  103 (Documents) |  |
|  | Executive summary/abstract |  |  |  |
| 5 | Provide a summary of the recommendations contained in the adapted guideline. | - Yes - No - Unclear | 66-( Summary  recommendations) |  |
|  | Abbreviations and acronyms |  |  |  |
| 6 | Define key terms and provide a list of abbreviations and acronyms (if applicable). | - Yes - No - Unclear | 5- (Abbreviations) |  |
|  | Contact information of the guideline adaptation group |  |  |  |
| 7 | Report the contact information of the developer of the adapted guideline. | - Yes - No - Unclear | 2-4 pages  (Authorship list)    1--Head Of EPG    2-Adaptation  Clinical  &Methodology Work Group.    3--External  (Reviewers for Validation Group. | Title Page of submission article includes Full information about  - Head of National  Egyptian Guideline  Committee  -Clinical work Group Members for  SSNS / SRNS  -Methodology  Group Members  -Reviewers local and international reviewers for |
|  | Scope |  |  |  |
|  | Source guideline(s) |  |  |  |
| 8 | Report the name and year of publication of the source guideline(s), provide the citation(s), and whether source authors were contacted. | - Yes - No - Unclear | 1(Cover Page)  5(Acknowledge)  96-100 (Methodology)  103(Documents)  104 (Appraisal)  107(permissions) | - referred to in recommend. Statements, figures, and tables.  -Listed in  methodology references p 120  -Source guidelines websites p 99, 103 |
|  | Brief description of the health problem(s) |  |  |  |
| 9 | Provide the basic epidemiological information about the problem (including the associated burden), health systems relevant issues, and note any relevant differences compared to the source guideline(s). | - Yes - No - Unclear | 9-20  (introduction  &background) | Included in  - Rationale of recommendations  Specially for adapted or added recommendations or practice points. eg *steroids vs steroid sparing in FR/SD.  *Daily VS alt day steroid in FR  -Special focus on this area is handled with examples inside discussion of the submitted article. |
|  | Aim(s) and specific objectives |  |  |  |
| 10 | Describe the aim(s) of the adapted guideline and specific objectives, and note any relevant differences compared to the source guideline(s). | - Yes - No - Unclear | -22 ( objectives) | -Proper initial diagnosis of NS in children  -Referral to pediatric  Nephrologist for extended investigations as needed.  - Proper treatment of initial episode and relapses.  -Maintain long remission for frequently relapsing and steroid dependent  --management of complications of disease or therapy  -Follow-up of pts for clinical and lab monitoring.  -family orientation with diet and activity, home assessment of proteinuria.  -Vaccination, what to do on contact with infected cases while on immunosuppressive drugs?  All previous objectives are fully discussed in our source guidelines, However in our area we need to add:  -Support referral to pediatric nephrologist  -Define red flags for referral  -Diagnosis workup to include infections especially of local epidemiology profile eg HBV, HCV, COVID, TB,  -Early Genetic testing in infantile, familial, syndromic  -Consider dug costs, availability, degree paeientt response in our area with reported local studies. [ see article discussion] |
|  | Target population(s) |  |  |  |
| 11 | Describe the target population(s) and subgroup(s) (if applicable) to which the recommendation(s) is addressed in the adapted guideline, and note any relevant differences compared to the source guideline(s). | - Yes - No - Unclear | 22 (PIPOH)  Model |  |
|  | End-users and settings | Assessment | Pages | Notes |
| 12 | Describe the intended target users of the adapted guideline, and note any relevant differences compared to the source guideline(s). | - Yes - No - Unclear | 22-24 (Scope  &purpose, PIPOH)  75 (implementation  consideration) |  |
| 13 | Describe the setting(s) for which the adapted guideline is intended, and note any relevant differences compared to the source guideline(s). | - Yes - No - Unclear | 22-24 (Scope & purpose, PIPOH)  75 (implementation consideration) |  |
|  | Rigor of development |  |  |  |
|  | Guideline adaptation group |  |  |  |
| 14 | List all contributors to the guideline adaptation process and describe their selection process and responsibilities. | - Yes - No - Unclear | 2-4 (Authorship group &Editorial Board) included GL Adaptation group &external review group with eminent clinical and methodology experts and including Authors of the CPG Article /Manuscript. | Submission Title page includes contributions. |
|  | Adaptation framework/methodology |  |  |  |
| 15 | Report which framework or methodology was used in the guideline adaptation process. | - Yes - No - Unclear | 9 (Adapted  ADAPTE Method  9 (Guideline Status)  74 (Adaptation  Process)  96-100 Figures & Steps of CPG adaptation Methodology)  120(References). | Methodology of submission article in appendix of  article |
|  | Source guideline(s) |  |  |  |
| 16 | Describe how the specific source guideline(s) was (were) selected. | - Yes - No - Unclear | 22 (Scope  Purpose)  96 (inclusion & exclusion criteria)  104(appraisal) | - Selection criteria in appendix (methodology documents.   - AGREE PLUSS  Critical appraisal  Documents in CPG & article appendix. |
|  | Key questions |  |  |  |
| 17 | State the key questions of the adapted guideline using a structured format, such as PICO (population, intervention, comparator, and outcome), or another format as appropriate | - Yes - No - Unclear | 22-24  96 | Quest p22  PIPOH Format p 22 |
| 18 | Describe how the key questions were developed/modified, and/or prioritized. | - Yes - No - Unclear | 22-24  96 | Quest p22    PIPOH Format p 22 |
|  | Source recommendation(s) |  |  |  |
| 19 | Describe how the recommendation(s) from the source guideline(s) was (were) assessed with respect to the evidence considered for the different criteria, the judgments and considerations made by the original panel. | - Yes - No - Unclear | 22-24    96-100 | Methodology adaptation steps in appendix steps |
|  | Evidence synthesis |  |  |  |
| 20 | Indicate whether the adapted recommendation(s) is/are based on existing evidence from the source guideline(s), and/or additional evidence | - Yes - No - Unclear | - 96-99  (Methodology)     - 80,81 (fig with   Graded evidence)       - 105-106 (Evidence grading for source Guidelines)   sources)       - 109   (References  list) | - Original   Recommendations from the source CPGs with their relevant evidence were accepted. Evidence from source GL fig p 80, 81     - Other sources for adapted modified statements or good practice points considering   evidence grade and level.  -References are listed in reference  list. p 109 |
| 21 | If new research evidence was used, describe how it was identified and assessed. | - Yes - No - Unclear | 109 | - Search in systematic reviews.  - High quality EB de novo guidelines on the web eg Asian guidelines 2022. or KDIGO12.  - Literature publications as Sub-Saharan  Africa studies or Canadian commentary on KDIGO 12 (see  article discussion), All are Included in reference list and are freely downloadable  from their official sites clearly stated at the end in reference list. |
|  | Assessment of the certainty of the body of evidence and strength of recommendation |  |  |  |
| 22 | Describe the approach used to assess the certainty/quality of the body/ies of evidence and the strength of recommendations in the adapted guideline and note any differences (if applicable) compared to the source guideline(s). | - Yes - No - Unclear | 75  (Methodology steps) | Recommendation for Three source guidelines (diagnosis or treatment) may be different in some areas.  Therefore:   - EPG selection for which to choose was based on preappraised Certainty or evidence strength, availability and acceptability of intervention, local limiting factors related to the health care |
|  | Decision-making processes |  |  |  |
| 23 | Describe the processes used by the guideline adaptation group to make decisions, particularly the formulation of recommendations. | - Yes - No - Unclear | 75 ( Adaptation steps  /Methodology ) | -We Adopted statements approved by the three CPGs that match disease profile in the Egyptian community and healthcare context and Applicable in our health settings.  -We adapted few to match disease profile in our community eg  * High rate of consanguinity, infections.  * Available facilities and expertise  * Drug costs  *pt compliance  *Local Health  system settings  *Medical  insurance system |
|  | Recommendations | Assessment | Pages | Notes |
|  | Recommendations |  |  |  |
| 24 | Report recommendations and indicate whether they were adapted, adopted, or *de novo*. | - Yes - No - Unclear | 74-75  24-33  (Recommendation statements ) | - Adopted from three source CPGs  - Added recommendation  s and practice points.  -Adapted modified statements with evidence or practice points.   - See submission   article discussion |
| 25 | Indicate the direction and strength of the recommendations and the certainty/quality of the supporting evidence and note any differences compared to the source recommendations(s) (if applicable). | - Yes - No - Unclear | 2433  (Recommendation statements , Rationale ) | - See submission article discussion.  - See Rationale of recommendations at guideline /  recommendations. p24-33 |
| 26 | Present separate recommendations for important subgroups if the evidence suggests important differences in factors influencing recommendations and note any differences compared to the source recommendations(s) (if applicable). | - Yes - No - Unclear | 24-33  (Recommendations statement, Rationale) | -See submission/ article discussion  -Rationale of  recommendations.  eg: First episode /FR/SD treatment |
|  | Rationale/explanation for recommendations |  |  |  |
| 27 | Describe the criteria/factors that were considered to formulate the recommendations or note any relevant differences compared to the source guideline(s) (if applicable). | - Yes - No - Unclear | 24-33 | - Rationale of all   recommendations  is discussing such factors with each statement, to support stakeholder decision towards Certain issues. |
|  | External review and quality assurance |  |  |  |
|  | External review |  |  |  |
| 28 | Indicate whether the adapted guideline underwent an independent external review. If yes, describe the process. | - Yes - No - Unclear | 73 | Local and international reviewers shared validation and finalization of the CPG full document. KDIGO assigned Dr Federica Zotta from Italy. 3 local professors of PN representing different universities who are known as international figures in PN.   - Disclosure   documents are included in guideline  appendix  -Interactive virtual meetings for focus group discussion of their comments with attendance of all work group members.  -Final draft included all reviewers’ comments.  -Dr Zotta revised two times and approved the final draft.  -All documents for online zoom meetings are available at EPG secretarial office. |
|  | Organizational approval |  |  |  |
| 29 | Indicate whether the adapted guideline obtained organizational approval. If yes, describe the process. | - Yes - No - Unclear |  | Apart from our National objectives to improve pt care,  we look for regional  dissemination in  Africa. our partnership with IPNA and KDIGO and hopefully readers of your journal will make  dissemination of recommendations more rewarding.  -Adapted guidelines using high quality source CPGs are very promising for low or limited resource countries like Egypt.  We obtained  official approval and permission from source guidelines developer organization to adapt their CPGs to the Egyptian health care context via email  communications, |
|  | Funding, declaration, and management of interest |  |  |  |
|  | Funding source(s) and funder role(s) |  |  |  |
| 30 | Report all sources of funding for the adapted guideline and source guideline(s), and the role of the funders. | - Yes - No - Unclear | 108 | no funding |
|  | Declaration and management of interests |  |  |  |
| 31 | Report all conflicts of interest of the adapted and the source guideline(s) panels, and how they were evaluated and managed. | - Yes - No - Unclear | 108 | no conflicts of interests for either adapted or source guidelines in this national adaptation project. All contributors are volunteering. |
|  | Other information |  |  |  |
|  | Implementation |  |  |  |
| 32 | Describe the potential barriers and strategies for implementing the recommendations (if applicable). | - Yes - No - Unclear | 75-78  (implementation  consideration)  101-103  (Implementation  strategy)      100-103  (where to refer? | Potential barriers include:  - Limited resources  - High-cost therapy  -poor medical insurance  - Poor facilities and expertise in many areas especially rural areas.  - Referral to expert secondary and tertiary care settings is lagging in countryside rural areas.  - Referral to pediatric nephrologist, Lab  immunology, genetic tests are not always available …  - poor infrastructure that hampers delivery of medicine to remote parts of the country.  - cultural factors with patient and doctor reluctance to use guidelines in some areas,  Therefore  Guideline  Implementation strategy (76-78) have been included in the draft p76,77,78 Documents for  sites of referral  (p 100-103) |
|  | Update |  |  |  |
| 33 | Briefly describe the strategy for updating the adapted guideline (if applicable). | - Yes - No - Unclear | 74 (update plan) | After three years of publication. (Published 2022)  (Update 2025), after checking for updates in source CPGs.  Except if any breakthrough evidence-based recommendations  are published before that date.  - Consultation of expert opinion in the field on changes needed to be conducted based on newest evidence, publications, clinical audit and feedback from local health settings in PIPOH model p 74 |
|  | Limitations and suggestions for further research |  |  |  |
| 34 | Describe the challenges of the adaptation process, the limitations of the evidence, and provide suggestions for future research. | - Yes - No - Unclear | 73 (Challenges in adaptation process) | Challenges in adaptation process include: -Search in literature for Clinical questions answers with evidenced statements related to PIPOH model. -Checking evidence grading in our source guideline when needed  -panel decision is both clinical and methodology considered  - Editing and formulating process.  - reviewing and peer reviewing, inter active discussions.  -Final drafting considering all reviewers comments.  All these items are included in guideline appendix, or National Guideline  Committee |

**Annals of Internal Medicine. March 2022, Vol. 175 No. 5. Doi: 10.7326/M21-4352 http://www.annals.org/**

**Methodology Reference**

Yang Song; Pablo Alonso-Coello; Monica Ballesteros; Francoise Cluzeau;Robin W.M. Vernooij; Thurayya Arayssi; Soumyadeep Bhaumik; Yaolong Chen, MMed; Davina Ghersi; Etienne V. Langlois; Paulina Fuentes Padilla; Holger J. Schünemann; Elie A. Akl; Laura Martínez García; and RIGHT-Ad@pt Working Group. A Reporting Tool for Adapted Guidelines in Health Care: The RIGHT-Ad@pt Checklist. Annals of Internal Medicine Vol. 175 No. 5. Doi : 10.7326/M21-4352

**IPNA 2020 & KDIGO 2021 Tables**

**Table (1): Definitions related to Nephrotic Syndrome in Children.**

**(IPNA 2020 and KDIGO 2021)**

| Term | Definitions |
| --- | --- |
| Nephrotic-range proteinuria | **UPCR ≥ 200 mg/mmol (2 mg/mg) in first morning void or**  **24 h urine sample ≥ 1000 mg/m2/day corresponding to 3+ or 4+**  **by urine dipstick.** |
| Nephrotic syndrome | **Nephrotic-range proteinuria and either hypoalbuminemia (serum albumin < 30 g/l) or edema when serum albumin level is not available.** |
| Complete remission | **UPCR (based on first morning void or 24 h urine sample) ≤ 20 mg/mmol (0.2 mg/mg) or negative or trace dipstick on three or more consecutive occasions.** |
| Partial remission | **UPCR (based on first morning void or 24 h urine sample) > 20 but < 200 mg/mmol and, if available, serum albumin ≥ 30 g/l.** |
| Relapse | **Relapse Recurrence of nephrotic-range proteinuria.**   - **In children, relapse is commonly assessed by urine dipstick and is thus defined as dipstick ≥ 3+ on 3 consecutive days, or**   **UPCR ≥ 200 mg/mmol (2 mg/mg) on a first morning urine sample, with or without reappearance of edema in a child who had previously achieved partial or complete remission.** |
| Confirmation Period | **Time period between 4 and 6 weeks from PDN initiation during which response to further oral PDN and/or pulses of iv MPDN and RAASi are ascertained in patients achieving only partial remission at 4 weeks.**   - **A patient achieving complete remission at 6 weeks is defined as a late responder.** - **A patient not achieving complete remission at 6 weeks although he had achieved partial remission at 4 weeks is defined as SRNS.** |
| SSNS | **Complete remission within 4 weeks of prednisone or prednisolone (PDN) at standard dose (60 mg/m2/day or 2 mg/kg/day, maximum 60 mg/day).** |
| Infrequent relapsing NS | **˂ 2 relapses per 6 months or ˂ 4 relapses per 12 months.** |
| Frequent relapsing NS | **≤ 2 relapses per 6 months or ≤ 4 relapses per 12 months.** |
| Steroid dependent NS | **Relapses during therapy with prednisone or prednisolone (either at full dose or during tapering) or within 15 days of prednisone or prednisolone discontinuation.** |
| SRNS | **Lack of complete remission within 4 weeks of treatment with PDN at standard dose.** |
| Late Responder NS | **Complete remission at 6 weeks.** |
| CNI-resistant SRNS | **Absence of at least partial remission after 6 months of treatment with a CNI at adequate doses and/or levels.** |
| Multi-drug-resistant SRNS | **Absence of complete remission after 12 months of treatment with 2 mechanistically distinct steroid-sparing agents at standard doses (see text).** |
| Secondary steroid resistance | **Children with initial steroid-sensitivity who in subsequent relapses develop SRNS.** |
|  |  |
| Table abbreviations | |
| UPCR urine protein/creatinine ratio, SSNS steroid sensitive nephrotic syndrome,  SRNS steroid-resistant nephrotic syndrome, PDN prednisolone or prednisone,  MPDN methylprednisolone, RAASi renin-angiotensin-aldosterone system, CNI calcineurin inhibitor | |

**Reproduced with permission from IPNA 2020 and KIDIGO 2021.**

**Table 2: Initial workup and follow-up for a child with steroid-resistant nephrotic syndrome (IPNA 2020).**

| Table 2 Pediatr Nephrol (2020) 35:1529–1561 | | |
| --- | --- | --- |
| Investigations | **Initial work up** | **Follow-up mentoring** |
| Clinical Evaluation |  |  |
| Patient history  – Including results of dipstick assessments at home, physical activity, fever episodes, pain, abdominal discomfort, swelling, fatigue, school attendance, adherence to medication, menstrual cycle in female adolescents  - Search for risk factors for secondary causes As appropriate (sickle cell disease, HIV, SLE, HepB, malaria, parvovirus B19)  - Check for tuberculosis in endemic areas before  starting immunosuppressant drugs | ✓  ✓  ✓ | **Every 3 months**  **As appropriate**  **As appropriate** |
| Physical examination  - Assessing fluid status including signs of edema (e.g., ascites, pericardial & pleural effusions), tetany, lymphadenopathy - Drug toxicity (e.g., eyes, skin) Every 3 months -  - Skeletal status - Extrarenal features, e.g., dysmorphic features  ambiguous genitalia  - Full neurological examination & standardized  assessment of cognitive status   - Pubertal status: Tanner stage, testicular volume in boys   ( in patients aged > 10 years)    - Vital parameters: blood pressure     - Anthropometry   Growth chart: height/length, weight, Head circumference < 2 years  Calculation of BMI and annual height velocity   - Vaccination status   Check and complete, especially for encapsulated bacteria— Pneumococcal, Meningococcal, Hemophilus Influenza, and Varicella-Zoster.   - Family history. - Renal and extrarenal manifestations. - Consanguinity | ✓  ✓  ✓  ✓  ✓ | **As appropriate**  **Every 3 months**  **Every 3 months**  **Every 3 months**  **As appropriate**  **Every 12 months or**  **as appropriate**  **Every 12 months**  **Every 3 months; yearly 24h ambulatory BP monitoring hypertension, if feasible.**      **Every 3 months (monthly in infants)**  **Every 12 month or as appropriate**  **Every 12 month or as appropriate** |
| Biochemistry |  |  |
| Urine  Spot urine (first morning void) or 24 h urine: protein/creatinine  Urinalysis including hematuria  Spot urine: calcium/creatinine ratio, low molecular weight proteinuria (e.g., α1-microglobulin/creatinine ratio    Blood  Complete blood count (CBC)  Creatinine, BUN, or urea  Electrolytes (including ionized calcium, potassium* and albumin corrected albumin if available)  Serum albumin, total protein  Blood gas analysis (HCO3)  C-reactive protein  Estimated GFR^b^  ALP, PTH, 25(OH) vitamin D  Lipid profile (LDL- and HDL-cholesterol, triglycerides)    Baseline coagulation tests (prothrombine time (INR),  aPTT, fibrinogen, ATIII), detailed thrombophilic screening in patients with reported previous thrombotic events, central venous lines, persistent nephrotic range proteinuria and/or increased familial history for thrombotic events.  Thyroid function (T3, FT4, TSH)  Immunoglobulin G      Glucose/fasting glucose  HbA1c  C3, antinuclear antibodies  ds-DNA, ENA, ANCA  HBs-Ag, anti-HCV-IgG, syphilis, and HIV tests Vaccination status including blood titer tests | **✓**  **✓**  **Conditional**  **✓**  **✓**  **✓**  **✓**  **✓**  **✓**  **✓**  **✓**  **✓**  **✓**  **✓**  **Conditional** | **Essential Every 3 months (more frequently until remission)**  **Every 6–12**  **Essential**  **Every 3 months (more frequently until remission)**  **and in CKD stage 4–5)**  **Every day or every other day when using high dose diuretics**    **As required (clinical decision)**  **Every 3 months (more frequently in CKD stage 4).**  **Every 12 months (more frequently in patients with CKD stages( 3- 5)**  **Every 12 months or as appropriate**  **At diagnosis and then as appropriate, e.g., in case of relapses.**  **Every 12 months or as appropriate especially in patients with prolonged proteinuria.**  **In case of recurrent infections**  **Every 6 months or as appro**  **Every 12 months or as appro**  **As appropriate**  **As appropriate**  **Before prednisolone & as app**  **Yearly or as appropriate** |
| Genetics |  |  |
| Next-generation sequencing (NGS)/Whole Exome  Sequencing (WES) | **✓** | **Extended screening for patients with SRNS depending on new findings (Table 3);**  **whole exome sequencing if indicated transplantation, if not previously performed.** |
| Drug-specific monitoring |  |  |
| CsA: and Tacrolimus: Drug trough levels | **Weekly during titration (for 4 weeks)** | **Thereafter every 3 months or as appropriate** |
| MMF: mycophenolic acid kinetic (2 h) c    Rituximab – CD19 B cell count: baseline  Statins: creatinine kinase (CK) – If on statins, every 6 months  Prolonged glucocorticoid therapy Conditional  Ophthalmological examination for cataract  and intraocular pressure  Bone mineral density by lumbar DEXA | **AUC after 4 weeks of treatment.**  **1 month after the first dose (nadir)**  **every 6 months**  **Conditional** | **Thereafter every 6–12 months or as appropriate.**  **Every 1–3 months until B cell recovery**  **every 6 months**  **Conditional** |
| Imaging |  |  |
| Renal ultrasound: renal echogenicity and size of kidneys | ✓ | **At presentation (mandatory prerenal biopsy)** |
| Ultrasound of abdomen & pleural space (ascites, effusions, thrombosis)    Cardiac ultrasound (left ventricular mass, effusions)  Chest X-ray    X-ray of the left wrist (bone age assessment  in children aged > 5 years, mineralization) | ✓  ✓  ✓  ✓ | **as appropriate**    **Every 12 months in hypertensive patients or in case of severe edema**  **Optional If indicated**    **Every 12 months or as appropriate** |
| Histopathological Renal Biopsy |  |  |
|  | ✓ | See text: at diagnosis, and subsequently if indicated: in case of unexplained drop in eGFR, unexplained increase in  proteinuria, to rule out and/or to monitor CNI nephrotoxicity during prolonged (< 2 years) treatment. |
| Dietary assessment |  |  |
| Dietician review and advice by a dietician  regarding salt, potassium, caloric and protein  intake | ✓ | **Every 3 months (more frequently in infants, malnourished patients, and patients with CKD stage 4–5)** |
| Assessment for extrarenal involvement |  |  |
| Depending on underlying disease and clinically evident extrarenal features: -  Brain MRI  (e.g., microcephaly, psychomotor delay, mental retardation, myoclonic epilepsy, tremor, ataxia, hypotonia)  Interdisciplinary evaluation by Ophthalmology  (e.g., microcoria, cataract, glaucoma, optic atrophy, keratoconus, macular spots, lenticonus, nystagmus).  Cardiology (e.g., congenital heart defects) | ✓  ✓  ✓ | **If indicated**  **If indicat**ed |

**Reproduced with permission from IPNA 2020.**

**Table 3- Steroid sparing therapy in SSNS (KDIGO 2021)**


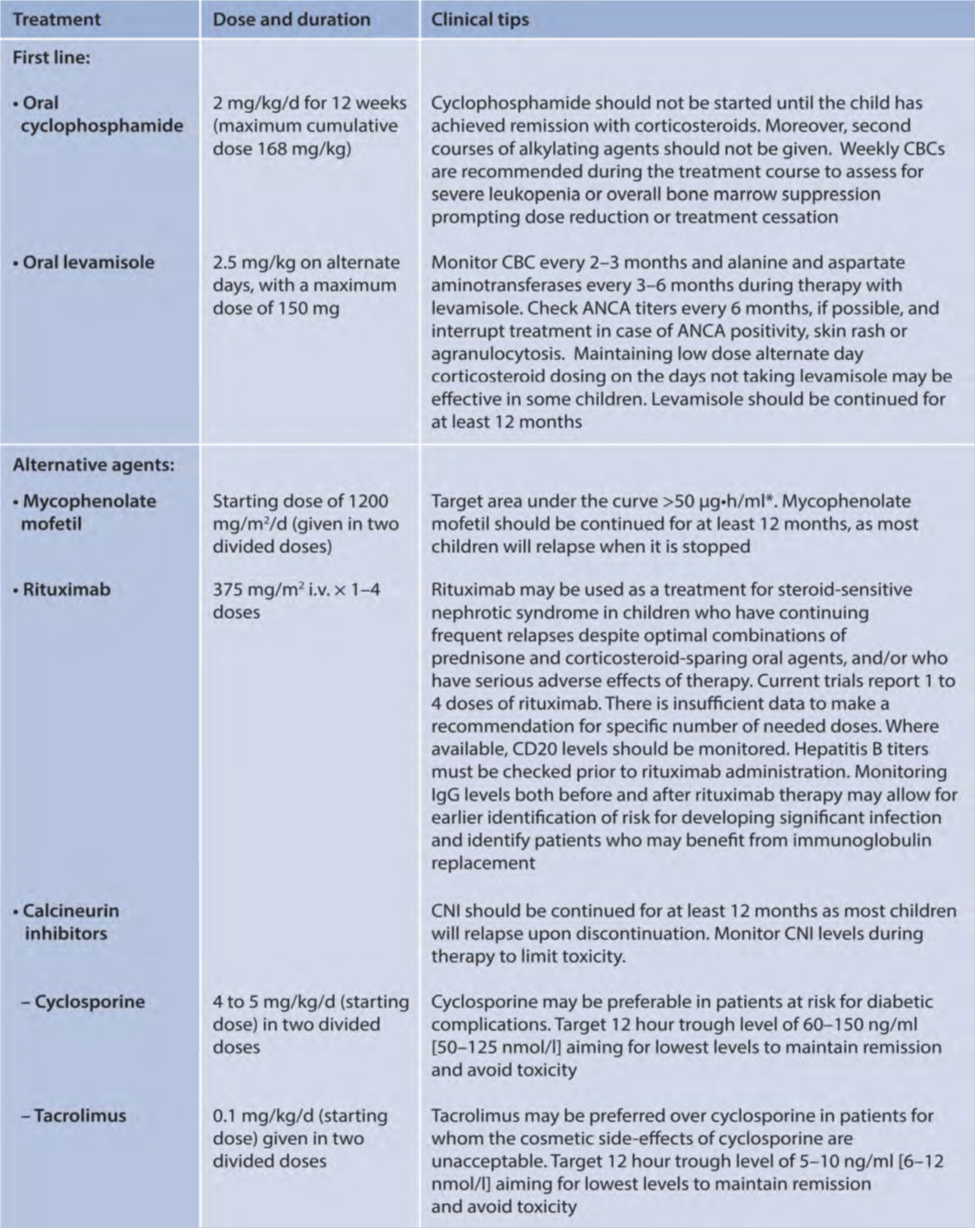


**Reproduced with permission from KDIGO 2021.**
